# Effect of appropriate empirical antimicrobial therapy on mortality of patients with Gram-negative bloodstream infections: a retrospective cohort study

**DOI:** 10.1186/s12879-023-08329-2

**Published:** 2023-05-23

**Authors:** Shanshan Xu, Zhihui Song, Furong Han, Chao Zhang

**Affiliations:** 1 Department of Pharmacy, Beijing Tongren Hospital, Capital Medical University, Beijing, 100730 China; 2 No.1 Dongjiaomin Lane, Beijing, Dongcheng District China

**Keywords:** Appropriate empirical antimicrobial therapy, Mortality, Bloodstream infections

## Abstract

**Background:**

Little evidence exists regarding the prevalence of pathogens in bloodstream infections (BSIs), the mortality risk, and the benefit of combination therapy over monotherapy. This study aims to describe patterns of empiric antimicrobial therapy, and the epidemiology of Gram-negative pathogens, and to investigate the effect of appropriate therapy and appropriate combination therapy on the mortality of patients with BSIs.

**Methods:**

This was a retrospective cohort study including all patients with BSIs of Gram-negative pathogens from January 2017 to December 2022 in a Chinese general hospital. The in-hospital mortality was compared between appropriate and inappropriate therapy, and between monotherapy and combination therapy for patients receiving appropriate therapy. We used Cox regression analysis to identify factors independently associated with in-hospital mortality.

**Results:**

We included 205 patients in the study, of whom 147 (71.71%) patients received appropriate therapy compared with 58 (28.29%) who received inappropriate therapy. The most common Gram-negative pathogen was Escherichia coli (37.56%). 131 (63.90%) patients received monotherapy and 74 (36.10%) patients received combination therapy. The in-hospital mortality was significantly lower in patients administered appropriate therapy than inappropriate therapy (16.33% vs. 48.28%, p = 0.004); adjusted hazard ratio [HR] 0.55 [95% CI 0.35–0.84], p = 0.006). In-hospital mortality was also not different in combination therapy and monotherapy in the multivariate Cox regression analyses (adjusted HR 0.42 [95% CI 0.15–1.17], p = 0.096). However, combination therapy was associated with lower mortality than monotherapy in patients with sepsis or septic shock (adjusted HR 0.94 [95% CI 0.86–1.02], p = 0.047).

**Conclusions:**

Appropriate therapy was associated with a protective effect on mortality among patients with BSIs due to Gram-negative pathogens. Combination therapy was associated with improved survival in patients with sepsis or septic shock. Clinicians need to choose optical empirical antimicrobials to improve survival outcomes in patients with BSIs.

**Supplementary Information:**

The online version contains supplementary material available at 10.1186/s12879-023-08329-2.

## Introduction

Bloodstream infections (BSIs) is a leading cause of high mortality in hospital. The short-term mortality of BSIs is ranging from 10 to 30% [1–3]. Early appropriate empirical antimicrobial therapy (AEAT) is associated with improved outcomes and inappropriate empirical antimicrobial therapy (IEAT) is associated with increased mortality in patients with BSIs [4–6]. However, the lag in obtaining the susceptibility of isolates as well as the rise of antibiotic resistance makes AEAT challenging.

Broad-spectrum antibiotics are recommended for patients with suspected severe infections [6, 7], but the widespread use of broad-spectrum antibiotics to all patients may increase antimicrobial resistance, *C. difficile* infection, antibiotic-related toxicities, and costs [8–10]. Although the initial use of combination therapy for Gram-negative bacteria is usually recommended in de-escalation strategies for serious infections, the advantages and disadvantages of combination therapy compared with monotherapy are controversial, and relevant studies have mainly been limited to bacteremia or specific infection. Therefore, we designed a retrospective study of patients with BSIs to examine the association between in-hospital mortality and AEAT using advanced methods in controlling confounding factors including patient-related factors like severity, comorbidities, source of infections, pathogen-associated factors like the type of Gram-negative pathogens and antibiotic resistance profile. We also analyzed whether or not the combination therapy directed against Gram-negative bacteria might be associated with lower in-hospital mortality in patients with BSIs, especially in patients with sepsis and septic shock.

## Study design and patients

This retrospective cohort study was conducted in a general hospital in China. We retrospectively analyzed all episodes of BSIs in adult (≥ 18 years) patients who received treatment with at least 1 new systemic antimicrobial agent within the initial 2 days of the blood sample collection, identified between Jan 1, 2017, and Dec 31, 2022. Exclusion criteria were missing key data, neutropenia, polymicrobial BSIs, death sooner than 24 h after BSIs onset, repeat BSIs episodes during the study period, special types of BSIs such as infective endocarditis and brain abscess, and active antimicrobials therapy for at least 48 h before blood culture.

### Data collection and definitions

The following data were extracted from the electronic medical records, including demographics, nosocomial or community acquisition, clinical and laboratory findings, comorbidities, the severity of the acute condition at presentation according to the Pitt bacteraemia score, microbiologic data, and source of infection. Cardiovascular disease was defined as the presence of one or more of the following conditions: previous coronary heart disease, peripheral arterial disease, heart failure, paroxysmal atrial fibrillation, and chronic (persistent or permanent) atrial fibrillation [11].

We also collected and analyzed the antimicrobial therapy regimens. All the antimicrobial therapy regimens were evaluated by an infectious disease specialist. We defined appropriate antimicrobial therapy as both appropriate timing and an appropriate antimicrobial regimen. We considered antimicrobial therapy timing appropriate if administered within optimal time windows (from index blood culture collection to administration of appropriate antimicrobial therapy), which were 24 h for Klebsiella pneumoniae bloodstream infection [12], 48.1 h for Enterococci bloodstream infection [13], 52 h for Pseudomonas aeruginosa (P aeruginosa) bloodstream infection [14], and 48 h for other pathogenic bacteria. The index culture was defined as the first blood sample collection with positive results during the hospitalization. An appropriate antibiotic regimen was defined as including at least one active drug against the offending pathogens based on in vitro susceptibility and with appropriate usage. If the regimen was changed during the course, we considered the antimicrobial regimen as the one started within optimal time windows after infection and administered for at least half of the duration of therapy [15]. BSIs episodes were classified as nosocomial and healthcare-associated [16]. The definition of sepsis and septic shock was defined according to the last proposed criteria (Sepsis-3) [17]. According to the National Healthcare Safety Network, the probable source of BSIs was classified using the following categories: BSIs from the central venous catheter (CVC), pulmonary infection, urinary tract infection (UTI), skin and soft tissue infection (SSTI), intra-abdominal infection (IAI), Bone or Joint infection (BJI), and primary BSIs in the absence of an identified source of infection growing the same organism as recovered from blood [18]. Multi-drug resistant (MDR) was defined as bacteria with resistance to 3 or more antimicrobial classes [19].

### Outcome and assessments

The primary outcome was the relationship between appropriate antibiotic therapy and in-hospital mortality. The secondary outcome was to evaluate if combination therapy was associated with lower in-hospital mortality. We defined combination therapy as therapy regimens including two or more in vitro active antibiotics administered at the same time and monotherapy as including only one drug.

### Statistical analysis

Categorical variables were described by counts and percentages, whereas continuous variables were expressed as mean ± standard deviation (SD) and medians and interquartile ranges (IQRs) according to their distribution. Continuous variables compared with Mann-Whitney U tests or Student t tests and categorical variables with χ²test or Fisher’s exact tests as appropriate. Kaplan-Meier survival analysis was used to compare the incidences of in-hospital mortality between AEAT and IEAT groups. We did multivariate analyses using Cox regression after assessing the proportional hazards assumption. We included variables with a univariate p of 0.2 or less for mortality and manually selected them in a backward stepwise manner according to their association and biological value. Hazard ratio (HR) and 95% confidence intervals (CIs) were calculated to evaluate the strength of any association.

In the sensitivity analysis, we examined appropriate empirical therapy use for different pathogens. We conducted additional sensitivity analysis by removing markers of disease severity (intensive care unit admission, vasopressor use, mechanical ventilation, and dialysis) from covariates because they might have been mediators in the associations. The level of significance was set at a P-value < 0.05 (two-sided). All the results were analyzed using a commercially available statistical software package (SPSS 23.0).

## Results

### Demographic and clinical characteristics

**Fig. 1:**
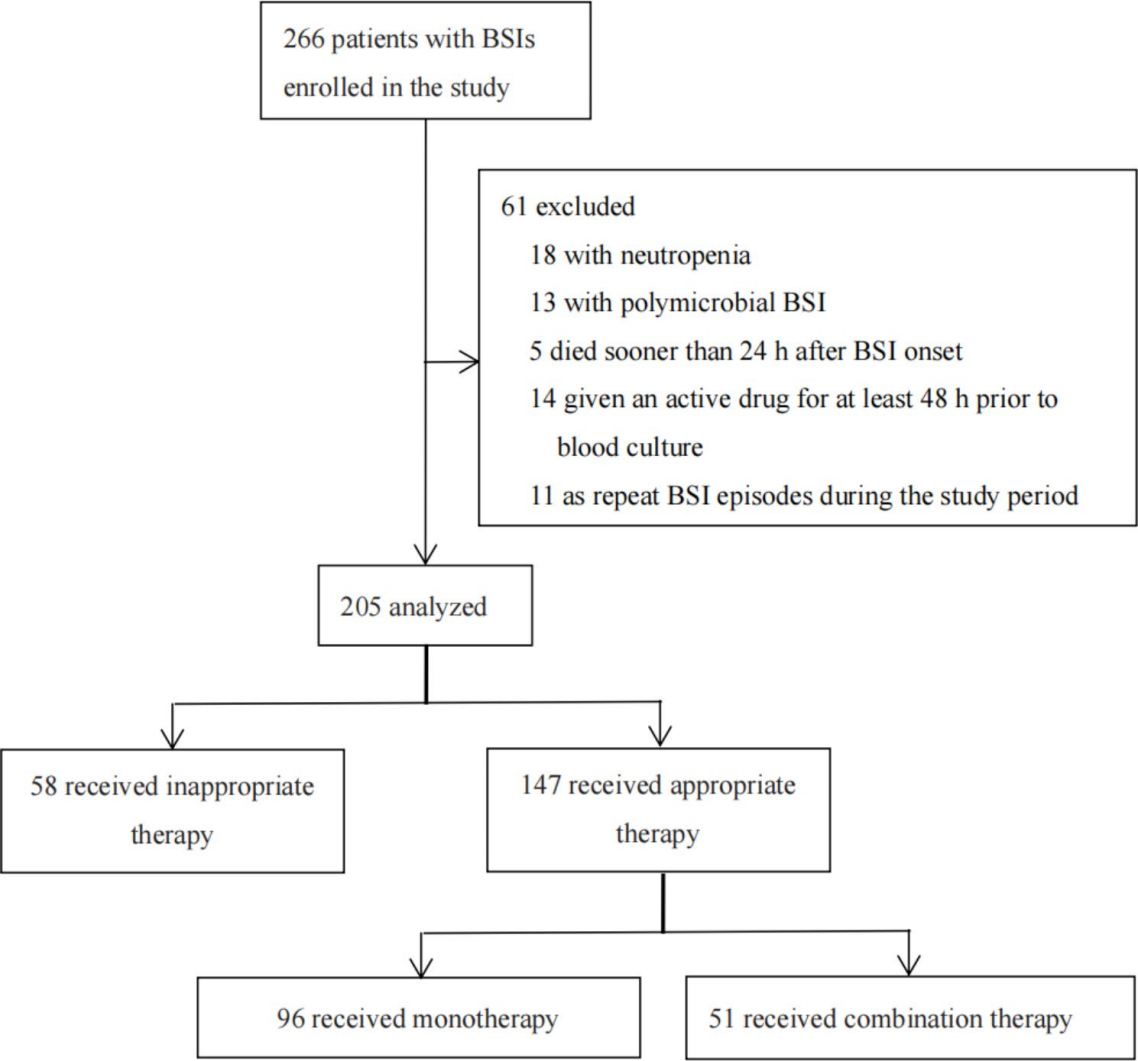
Flow chart of included patients with bloodstream infections

266 patients with BSIs were identified during the study period. 205 patients met the study eligibility criteria and were included in the study (Fig. 1). Table 1 summarizes the clinical and demographic characteristics of the study. The median age was 67 years (IQR 54–79) and 50.73% were male. The common sites of infection were urinary tract (24.39%), pulmonary (23.90%), and intra-abdominal (23.41%). Diabetes (34.15%) and cardiovascular disease (29.27%) were the most common comorbidities. Septic shock occurred in 33.66% of patients. 82.44% of BSIs were not healthcare-associated infections.

**Table 1:** Clinical and demographic characteristics of patients with BSIs receiving appropriate and inappropriate therapy

	All patients (n = 205)	Appropriate therapy (n = 147)	Inappropriate therapy (n = 58)	p value
Age, years, median (IQR)	67 (54–79)	66(54–79)	70.5(54.75.5–80)	0.589
Male sex	104(50.73%)	69(46.94%)	35(60.34%)	0.057
Comorbidities				
Cardiovascular disease	60(29.27%)	41(27.89%)	19(32.76%)	0.299
Diabetes	70(34.15%)	52(35.37%)	18(31.03%)	0.337
Chronic kidney disease	27(13.17%)	21(14.29%)	6(10.34%)	0.308
Chronic liver disease	6(2.93%)	4(2.72%)	2(3.45%)	0.545
COPD	6(2.93%)	3(2.04%)	3(5.17%)	0.222
Solid cancer	28(13.66%)	22(14.97%)	6(10.34%)	0.266
Immunosuppression	14(6.83%)	11(7.48%)	3(5.17%)	0.403
Site of infection				
Pulmonary	49(23.90%)	33(22.45%)	16(27.59%)	0.437
Intra-abdominal	48(23.41%)	36(24.49%)	12(20.69%)	0.563
Vascular catheter	14(6.83%)	7(4.76%)	7(12.07%)	0.062
Urinary tract	50(24.39%)	41(27.89%)	9(15.52%)	0.063
Skin or soft tissue	19(9.27%)	16(10.88%)	3(5.17%)	0.204
Bone or Joint	9(4.39%)	6(4.08%)	3(5.17%)	0.731
Primary BSI	16(7.80%)	8(5.44%)	8(13.79%)	0.045
Charlson comorbidity index score	1(0–2)	0(1–2)	0(1–2)	0.556
Pitt bacteraemia score	1(0−3.5)	1(0–3)	1(0–4)	0.480
SOFA Score on Culture Day	3(0−6.5)	2(0–6)	3(0–7)	0.760
APACHE II score	17(12−21.75)	17(12.25–21.75)	17(9.5–21.5)	0.262
ICU admission	112(54.63%)	77(52.38%)	35(60.34%)	0.191
Length of ICU stay after BSI, days	12(6.25–26.75)	9.5(5–21)	19.5(10.25–32.75)	0.038
Hospital length of stay after BSI, days	22(14–37)	19(13–32)	30(15.75–41.25)	0.033
Type of acquisition				0.006
Healthcare-associated	36(17.56%)	19(12.93%)	17(29.31%)	
Nosocomial	169(82.44%)	128(87.07%)	41(70.69%)	
Mechanical ventilation	50(24.39%)	25(17.01%)	25(43.10%)	<0.001
AKI	25(12.20%)	12(8.16%)	13(22.41%)	0.007
Septic shock	69(33.66%)	48(32.65%)	21(36.21%)	0.372

### Pathogen characteristics

The distribution of pathogens in patients with BSIs is summarized in Figure S1. Overall, Escherichia coli (E coli) was the most common pathogen (77/205, 37.56%), followed by Klebsiella species (52/205, 25.37%) and P aeruginosa (25/205, 12.20%). Of all the 205 patients included in the study, Ceftriaxone-resistant Gram-negative organisms (CTX-RO) were isolated in 20.98% (43/205), extended-spectrum beta-lactamase (ESBL) in 12.20% (25/205), and Carbapenem-resistant Enterobacteriaceae (CRE) in 8.78% (18/205). The proportions of AEAT varied from 38.89 to 89.61% for patients according to different pathogens and CRE had the lowest proportion. The proportions of AEAT for acinetobacter species and P aeruginosa were generally lower than other Gram-negative organisms.

### Associations between appropriate empirical antimicrobial therapy and in-hospital mortality

The proportions of appropriate therapy were 71.71% (147/205) compared with 28.29% (58/205) of inappropriate therapy. Of all the 91 patients with MDR bacteria infection, 33 (36.26%) patients received IEAT compared with 25 (21.93%) patients in the 114 patients with non-resistant bacteria infection. Patients with resistant pathogens were more likely to receive IEAT (p = 0.024). Comparison in the characteristics of patients who received inappropriate and appropriate therapy are shown in Table 1. 28 (48.28%) of 58 patients receiving IEAT died of all causes in the hospital compared with 24 (16.33%) of 147 receiving AEAT (absolute difference 31.95%, p = 0.004). The Kaplan-Meier curve for survival is shown in Figure S2 (log-rank p = 0.04). Univariate and multivariate analyses of in-hospital mortality are shown in Table 2. Appropriate therapy was independently associated with a protective effect (adjusted HR 0.55 [(0.35–0.84)], p = 0.006). Other factors independently associated with the overall in-hospital mortality, by Cox regression analysis, were SOFA score on culture day (1.08 [95% CI 1.03–1.12], p = 0.03), Pitt bacteremia score (HR 1.16 [95% CI 1.00–1.35], p = 0.048), source other than urinary tracts (HR 1.13 [95% CI 0.88–1.28], p = 0.049), sepsis or septic shock (HR 3.58 [95% CI 2.03–6.57], p = 0.001) and age (HR 1.03 [95% CI 1.01–1.05], p = 0.005).

**Table 2:** Univariate and multivariate Cox regression analyses for in-hospital mortality of patients with BSIs

	Crude analysis		Adjusted analysis	
	OR (95% CI)	P value	OR (95% CI)	P value
Age (per year)	1.04(1.02–1.06)	<0.001	1.03(1.01–1.05)	0.005
Male sex	1.03(0.91–1.18)	0.625	…	…
Nosocomial acquisition	0.88(0.45–1.72)	0.714	…	…
Source other than urinary tracts	1.15(0.97–1.37)	0.004	1.13(0.88–1.28)	0.049
ICU admission	1.19(1.01–1.39)	0.04	…	…
Charlson comorbidity index score (per unit)	1.04(1.02–1.06)	0.003	…	…
Mechanical ventilation	1.35(1.08–1.70)	0.009	…	…
Sepsis or septic shock	3.66(2.06–6.50)	<0.001	3.58(2.03–6.57)	0.001
Pitt bacteraemia score (per unit)	1.16(1.05–1.27)	0.003	1.16(1.00-1.35)	0.048
Appropriate therapy	0.55(0.36–0.82)	0.004	0.55(0.35–0.84)	0.006
MDR	1.04(0.88–1.22)	0.651	…	…
SOFA Score on Culture Day (per unit)	1.07(1.01–1.13)	0.031	1.08(1.03–1.12)	0.03

**Fig. 2:**
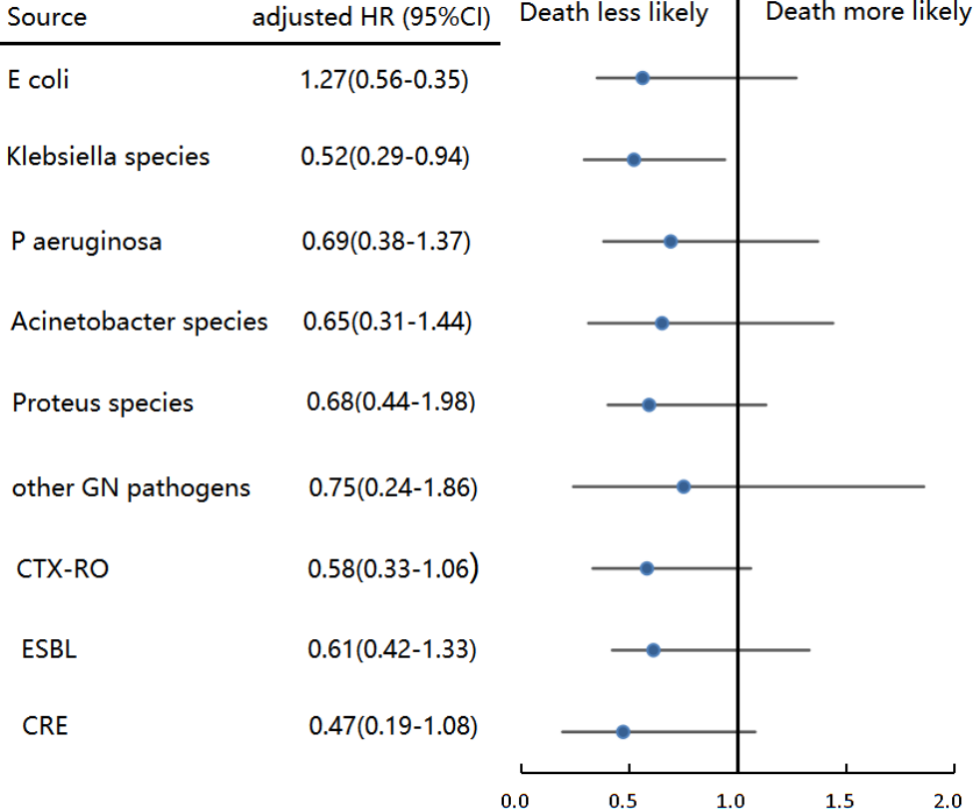
Adjusted Hazards of in-hospital death associated with appropriate empirical antimicrobial therapy by pathogen

In the sensitivity analysis, we removed markers of disease severity from covariates and the results showed that the appropriate therapy was also a protective effect for mortality (HR 0.50 [95% CI, 0.28–0.88], p = 0.017]). Compared with IEAT, AEAT was associated with lower in-hospital mortality for all the pathogens (Fig. 2).

### Associations between combination therapy and in-hospital mortality

Of all the 205 patients, 131 (63.90%) patients received monotherapy and 74 (36.10%) patients received combination therapy. The antimicrobials administered and their associated mortality is shown in Table S1. The most frequent drugs used in monotherapy were Cefoperazone-sulbactam and meropenem or Imipenem (Carbapenems). In combination regimens, Carbapenem and third-generation Cephalosporin were the most common. We built a multivariate Cox logistic regression model and reclassified the variables into inappropriate therapy, appropriate monotherapy, and appropriate combination therapy. Monotherapy (adjusted HR 0.54 [95% CI 0.42–0.77], p = 0.024) and combination therapy (adjusted HR 0.41 [95% CI 0.25–0.49], p = 0.001) both had a protective effect on in-hospital mortality.

Of the 147 patients who received appropriate therapy, 96 (65.31%) received monotherapy and 51 (34.69%) received combination therapy. The characteristics are shown in Table 3. 17 (17.71%) of 96 patients receiving monotherapy died of all causes in the hospital compared with 7 (13.73%) of 51 receiving combination therapy (absolute difference 3.98%, p = 0.534).

**Table 3:** Characteristics of patients with appropriate therapy receiving monotherapy or combination therapy

	Monotherapy (n = 96)	Combination Therapy (n = 51)	p value
Age, years, median (IQR)	66(50.25−78)	66(57.5–80)	0.151
Male sex	41(42.71%)	28(54.90%)	0.159
Comorbidities			
Cardiovascular disease	25(26.04%)	16(31.37%)	0.493
Diabetes	32(33.335)	20(39.22%)	0.478
Chronic kidney disease	13(13.54%)	7(13.73%)	0.975
Chronic liver disease	2(2.08%)	2(3.92%)	0.514
COPD	0	3(5.88%)	0.016
Solid cancer	14(14.58%)	6(11.76%)	0.635
Immunosuppression	5(5.21%)	6(11.76%)	0.15
Site of infection			
Pulmonary	19(19.79%)	14(27.45%)	0.289
Intra-abdominal	24(25%)	12(23.53%)	0.844
Vascular catheter	2(2.08%)	5(9.8%)	0.036
Genitourinary	33(34.38%)	8(15.69%)	0.016
Skin or soft tissue	7(7.29%)	1(1.96%)	0.175
Bone or Joint	5(5.21%)	9(17.65%)	0.014
Other	6(6.25%)	2(3.92%)	0.554
Charlson comorbidity index score	1(0–2)	1(0–2)	0.757
Pitt bacteraemia score	1(0–2)	2(1–4)	0.2
SOFA Score on Culture Day	1.5(0–5)	3(1–10)	0.005
APACHE II score	16(11.5–20)	16(11.75–21.25)	0.2
ICU admission	41(42.71%)	36(70.59%)	0.001
Length of ICU stay after BSI, days	7(4.5–11.5)	18(8–31)	<0.001
Hospital length of stay after BSI, days	12(8−16.5)	16(12–28)	0.362
Type of acquisition			0.467
Healthcare-associated	11(11.46%)	8(15.69%)	
Nosocomial	85(88.54%)	43(84.31%)	
Mechanical ventilation	10(10.42%)	15(29.41%)	0.004
AKI	6(6.25%)	6(11.76%)	0.245
Septic shock	25(26.04%)	23(45.10%)	0.019

We also did univariate and multivariate Cox regression analyses of in-hospital mortality in patients receiving AEAT (Table S2). Combination therapy was not associated with in-hospital mortality (adjusted HR 0.42 [95% CI 0.15–1.17], p = 0.096). But the interaction of sepsis or septic shock with combination therapy was protective (adjusted HR 0.94 [95% CI 0.86–1.02], p = 0.047), meaning that combination therapy was protective only in patients with sepsis or septic shock.

## Discussion

This study showed that approximately one of every three patients with Gram-negative BSIs received IEAT and IEAT was associated with increased in-hospital mortality in patients with BSIs. In our study, 48.28% of patients with IEAT died in the hospital. The in-hospital mortality rate of IEAT reported in previous studies varied from 13.6 to 68.3% according to different pathogens and the severity of the disease [7–13]. Timely antimicrobial therapy is critical to the prognosis in patients with BSIs. The administration time of antimicrobials varied with different pathogens and populations. The previous research demonstrated that optimal appropriate antimicrobial therapy time windows were 24 h for Klebsiella pneumoniae bloodstream infection [12], 48.1 h for Enterococci bloodstream infection [13], and 52 h for Pseudomonas aeruginosa bloodstream infection [14]. In our study, we adopted the above standards as AEAT. Our study demonstrated that appropriate administration of active therapy timely was associated with lower mortality no matter the severity of infection or the types of Gram-negative pathogens. The results were consistent with previous studies [7, 20, 21]. Several studies showed that IEAT was not associated with mortality and it might be explained by the sample size of the studied population or the definition of IEAT, et al. We also found that patients with MDR pathogens received IEAT more than 1.5 times as often compared with patients with non-resistant pathogens. But we did not find an association between antibiotic-resistant pathogens and mortality after adjusting for baseline and clinical characteristics as well as appropriate empiric antimicrobial therapy. The result was similar to another study [7].

It is controversial that whether or not combination therapy is associated with lower mortality. Although many studies found the survival benefits of combination therapy over monotherapy in patients with gram-negative bacteremia [22–25], some others did not [26, 27]. In some, the survival benefit of combination therapy was only showed in patients with gram-negative bacteremia caused by multidrug-resistant bacteria or *Pseudomonas* spp [28, 29]. A meta-analysis showed that combination antibiotic therapy improved survival and clinical response of high-risk, life-threatening infections, particularly those associated with septic shock but may be detrimental to low-risk patients [30]. Study-related adverse events were also more common in patients treated with combination therapy. Our results confirmed also that combination therapy is only associated with lower mortality for patients with sepsis or septic shock. The most common combination therapy regimens for patients with sepsis or septic shock were Carbapenem or Third-generation cephalosporin combined Quinolone. A retrospective cohort of patients with BSIs due to Carbapenemase-producing Enterobacteriaceae (CPE) also demonstrated that combination therapy was associated with improved survival of patients receiving appropriate therapy with a high pretreatment probability of death as measured by the modified INCREMENT-CPE mortality score compared with monotherapy [31]. These findings are important for the rational use of antibiotic for BSIs to reduce consumption of some drugs, relevant drug adverse events, and what’s more, bacterial drug resistance.

We found patients in the study received a lot of unnecessary broad empiric treatments, especially Carbapenems. The administration proportion of Carbapenems was almost 40% in the study. The guideline advised that initial broad-spectrum therapy was only for critically-ill patients with suspected sepsis and septic shock [32]. The frequent used Carbapenems may contribute to Carbapenem-resistant Enterobacteriaceae. A cohort study found unnecessarily broad empiric therapy was associated with a 26% increased risk of *C difficile* infection and more acute kidney injury [10]. We also observed patients received a lot of unnecessary treatments to treat GNB especially Vancomycin or Linezolid. The combined administration proportion with Vancomycin or Linezolid was more than 20% in the study. A meta-analysis [33] showed that the combination of Vancomycin was associated with renal toxicity. Therefore, to avoid overtreatment, it is a challenge for clinicians to evaluate whether empiric antimicrobial therapy is appropriate or not. The progressive advances in microbiological techniques have led to a shortening of time for the positivity of cultures, with the majority of blood cultures becoming positive within the first 24 h [34]. This could be helpful for physicians to rapidly evaluate the appropriateness of empiric antimicrobial therapy to avoid IEAT.

Our study has limitations. First, the retrospective study was performed at a single center and the sample is relatively small. Second, the resistance and antibiotic susceptibility of Gram-negative bacteria varied from hospital to hospital, and the results of the study may not be applicable to other hospitals. Third, we could not compare mortality in different combinations of antimicrobials with monotherapy because of the low numbers of patients in this subgroup, so that large sample of data is expected to do further analysis. Fourth, the exclusion of polymicrobial BSIs may have impacted the estimates. The strength of this study is the inclusion of different Gram-negative bacteremia and strict definitions and advanced methods in controlling for confounding factors.

## Conclusions

In the retrospective cohort study, we found that appropriate empiric antimicrobial therapy was associated with lower mortality in adult patients with BSIs. The combinations of antimicrobials were associated with lower mortality for patients with sepsis or septic shock. Unnecessary broad empiric antibiotics and unnecessary combination antibiotics were frequently prescribed. In the future, with the rapid development of diagnostic testing technologies for pathogens and their resistance or measurement of antibiotic blood concentrations [35], it is a challenge for clinicians to identify the best-tailored antimicrobial to improve outcomes in patients with BSIs.

## Electronic supplementary material

Below is the link to the electronic supplementary material.

Supplementary Material 1

## Data Availability

The datasets used and/or analysed in the study are available from the corresponding author on reasonable request.
