# Supplementary material for: Effect of appropriate empirical antimicrobial therapy on mortality of patients with Gram-negative bloodstream infections: a retrospective cohort study
Source: BMC Infect Dis. 2023 May 23;23:344. doi: 10.1186/s12879-023-08329-2 (PMC10204198; doi:10.1186/s12879-023-08329-2)
Supplement: Supplementary file 1 — Supplementary Material 1 [file 12879_2023_8329_MOESM1_ESM.docx]

**Table S1. In-hospital mortality of patients with bloodstream infections receiving different antimicrobial regimens**

|  | **All patients**  **(n=205)** | **Appropriate therapy**  **(n=147)** | **Inappropriate therapy**  **(n=58)** |
| --- | --- | --- | --- |
| Monotherapy |  |  |  |
| β-lactam | 14/118(11.86%) | 7/88(7.95%) | 7/30(23.33%) |
| Carbapenem | 3/36(8.33%) | 2/31(6.45%) | 1/5(20%) |
| Ceftazadime | 1/10(10%) | 1/8(12.5%) | 0/2 |
| Cefoperazone-Sulbactam | 3/42(7.14%) | 1/33(3.03%) | 2/9(22.22%) |
| Piperacillin-Tazobactam | 4/15(26.67%) | 2/9(22.22%) | 2/6(33.33%) |
| Ceftriaxone | 0/7 | 0/5 | 0/4 |
| Cefmetazole or Cefoxitin | 2/4(50%) | 1/2(50%) | 1/2(50%) |
| Aztreonam | 1/2(50%) | 0/0 | 1/2(50%) |
| Quinolone | 0/13 | 0/8 | 0/5 |
| Levofloxacin | 0/8 | 0/6 | 0/2 |
| Moxifloxacin | 0/5 | 0/2 | 0/3 |
| Combination therapy |  |  |  |
| Carbapenem included | 9/32(28.13%) | 6/21(28.57%) | 3/11(27.27%) |
| Third-generation cephalosporin included | 5/31(16.13%) | 5/23(21.74%) | 0/8 |
| Tazobactam or Sulbactam included | 4/29(13.79%) | 4/23(17.39%) | 0/6 |
| Tigecycline included | 3/11(27.27%) | 1/4(25%) | 2/7(28.57%) |
| Quinolone included | 3/14(21.43%) | 2/11(18.18%) | 1/3(33.33%) |
| Aminoglycosides included | 2/6(33.33%) | 1/4(25%) | 1/2(50%) |

**Table S2 Univariate and multivariate Cox regression analyses of variables associated with in-hospital mortality in patients receiving appropriate therapy, including severe sepsis or septic shock and its interaction with combination therapy**

|  | **Crude analysis** | | **Adjusted analysis** | |
| --- | --- | --- | --- | --- |
|  | **HR (95% CI)** | **P value** | **HR (95% CI)** | **P value** |
| Age (per year) | 1.02(1.00-1.05) | 0.111 | . . . | . . . |
| Male sex | 1.82(0.79-4.23) | 0.163 | . . . | . . . |
| Nosocomial acquisition | 1.83(0.43-7.84) | 0.414 | . . . | . . . |
| Source other than urinary or biliary tracts | 1.07(0.72-1.58) | 0.737 | . . . | . . . |
| ICU admission | 1.25(0.87-1.80) | 0.225 | . . . | . . . |
| Charlson comorbidity index score (per unit) | 1.02(0.86-1.20) | 0.846 | . . . | . . . |
| Mechanical ventilation | 1.34(0.78-2.30) | 0.297 | . . . | . . . |
| Severe sepsis or septic shock | 1.32(0.88-1.98) | 0.018 | 1.54(0.97-2.13) | 0.003 |
| Pitt bacteraemia score (per unit) | 1.26(1.10-1.43) | 0.001 | . . . | . . . |
| Combination therapy | 0.29(0.12-0.70) | 0.006 | 0.42(0.15-1.17) | 0.096 |
| MDR | 0.94(0.42-2.11) | 0.872 | . . . | . . . |
| SOFA Score on Culture Day | 1.03(0.95-1.12) | 0.467 | . . . | . . . |
| Interaction of  severe sepsis or septic shock with  combination therapy | . . . | . . . | 0.94(0.86-1.02) | 0.047 |


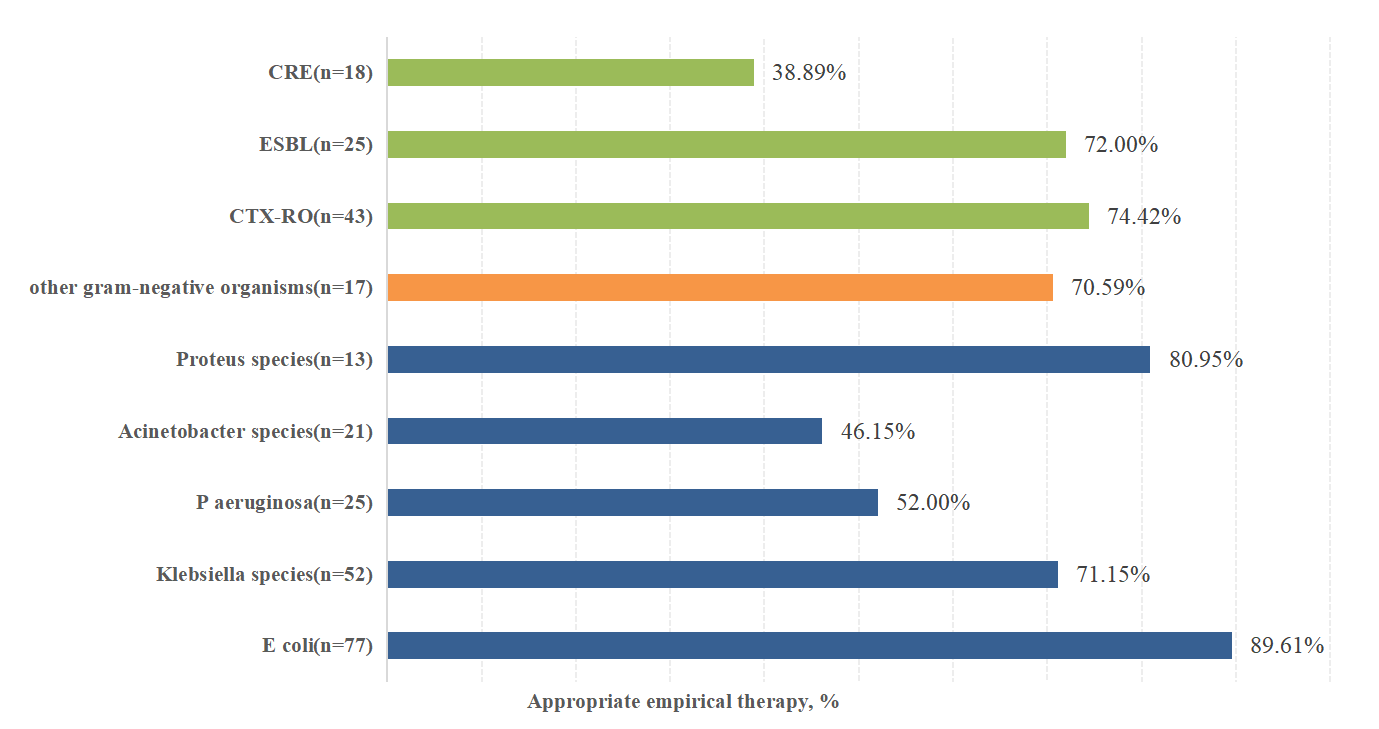


**Figure S1** Number of patients infected with pathogens and proportions of appropriate empirical antimicrobial therapy by pathogen


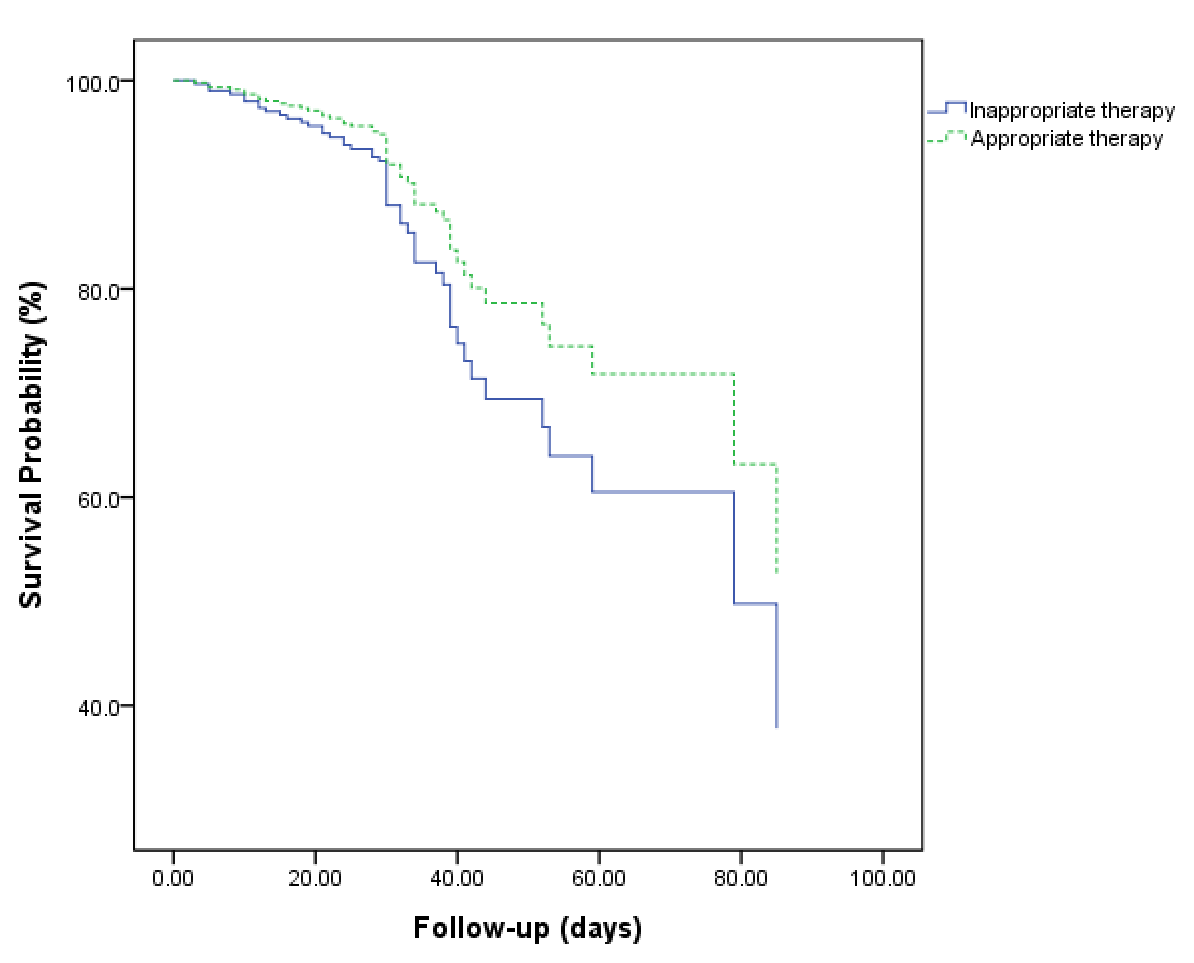


**Figure S2** Kaplan-Meier curves for in-hospital mortality in patients who received appropriate or inappropriate therapy
